# Gendered Cycles of Sexual Objectification: The Roles of Social Dominance Orientation and Perceived Social Mobility

**DOI:** 10.1007/s10508-024-03065-3

**Published:** 2024-12-19

**Authors:** Rheal S. W. Chan, Kai-Tak Poon

**Affiliations:** Department of Psychology and Centre for Psychosocial Health, The Education University of Hong Kong, Hong Kong, 999077 China

**Keywords:** Sexual objectification, Social dominance orientation, Social mobility, Gender, Victimization, Perpetration

## Abstract

Despite the high prevalence of sexual objectification, the understanding of gender differences in its victimization and perpetration remains limited. We bridged victim and perpetrator perspectives, expecting that objectification victimization positively predicts perpetration, and investigated the mediating role of social dominance orientation (SDO), and gender and perceived social mobility as moderators. Participants (valid *N* = 530) completed measures of sexual objectification victimization, perceived social mobility, SDO, and sexual objectification perpetration. We found that sexual objectification victimization predicted its perpetration, and that this relationship was stronger among men than women. SDO partially mediated the moderation effect of gender, whereby mediation through SDO was significant among men, but not significant among women. Moreover, a three-way interaction between sexual objectification victimization, gender, and perceived social mobility predicted SDO and carried subsequent implications for sexual objectification perpetration. Despite the mediation effect through SDO not achieving significance among women, it was significantly moderated by perceived social mobility. Specifically, the indirect effect among women with high perceived social mobility was significantly different than that among women with low perceived social mobility, but not significantly different than that among men, whose results were not influenced by perceived social mobility. This study provides a more nuanced understanding of gender in sexual objectification—specifically that gender differences may be related to social power and differences in socialization. The findings offer implications for the development of theories and clinical programs for coping with objectification victimization and preventing perpetration.

**Supplementary Information:**

The online version contains supplementary material available at 10.1007/s10508-024-03065-3.

## Introduction

Sexual objectification, defined as the reduction of a person to only their appearance or sexual function (Fredrickson & Roberts, 1997), has been suggested to be experienced by 75% of women, from leering to unwanted touching, at least once a week (Holland et al., 2017). It has been associated with numerous negative consequences, including increased trauma symptoms (Miles-McLean et al., 2015), anxiety and depression (Jiang et al., 2022; Szymanski, 2020), and antisocial responses (Poon & Jiang, 2020; Poon et al., 2023). Given the prevalence and impacts of sexual objectification, a more comprehensive understanding of its outcomes and factors which predict its perpetration is incredibly important. Despite men reporting victimization by sexual objectification (Brinkman & Rickard, 2009; Davids et al., 2019) and women reporting perpetrating sexual objectification (Gervais et al., 2018), most research on this phenomenon has focused on women as targets and men as perpetrators (e.g., Holland et al., 2017; Sáez et al., 2019). Thus, the consequences of men’s victimization and antecedents of women’s perpetration remain relatively unknown.

**Fig. 1:**
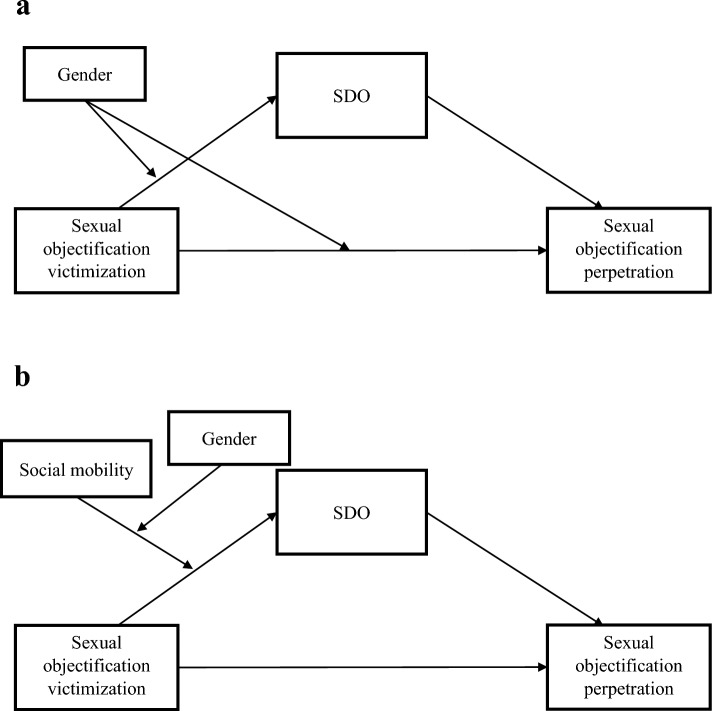
**a** Conceptual model for H3. **b** Conceptual model for H5

Research thus far has tended to consider sexual objectification victimization and perpetration in isolation (see Loughnan & Pacilli, 2014 for a review), however past research has shown that interpersonal victimization may lead to perpetration of the same maltreatment (Falla et al., 2022; Lee et al., 2016). Therefore, we aimed to bridge the victimization and perpetration perspectives to investigate whether sexual objectification victimization is associated with its perpetration, crucially considering this relationship among both men and women. Because sexual objectification has been demonstrated to be influenced by existing gender hierarchies (Bareket & Shnabel, 2020; Walton & Pedersen, 2022), we focused on factors related to social hierarchy and social status to understand the relationship between sexual objectification victimization, perpetration, and gender. Specifically, we proposed social dominance orientation (SDO), which is individuals’ preference for social hierarchies (Pratto et al., 1994), as a potential mechanism underlying the gender differences in the link between victimization and perpetration (see Fig. 1a for the conceptual model). We further considered perceived social mobility, referring to individuals’ beliefs about their chances for social status change and movement within a given social hierarchy (Day & Fiske, 2019), as a moderator. In particular, we predicted a three-way interaction between sexual objectification victimization, gender, and perceived social mobility in predicting SDO, with subsequent implications for sexual objectification perpetration in a moderated mediated moderation (see Fig. 1b for the conceptual model).

### Sexual Objectification and Gender

When individuals are targeted by any form of interpersonal victimization, they may have a greater sense of entitlement to behave selfishly to obtain benefits that their victimization denied them and avoid further negative outcomes (Poon et al., 2013; Zitek et al., 2010). Moral disengagement theory suggests that individuals targeted by interpersonal victimization may adopt the view that the form of interpersonal victimization experienced is normalized and acceptable (Bandura, 2002), in turn, becoming more likely to perpetrate the same form of victimization against others (Falla et al., 2022; Lee et al., 2016). Thus, sexual objectification victimization may predict the perpetration of sexual objectification.

However, gender may moderate the relationship between victimization and perpetration given gender differences in sexual objectification (Murnen & Smolak, 2000). Gender differences related to sexual objectification have been suggested to stem from gender socialization processes (Pecini et al., 2023; Vaes et al., 2013). Socialization in a society where men possess more social power may grant them the ability to face less consequences following antisocial behavior compared to women (Rudman & Glick, 2021). This is furthered by overwhelmingly reinforced gender roles that emphasize female-victim and male-perpetrator scripts as the norm in relation to sexual objectification (Dill & Thill, 2007; Endendijk et al., 2022).

Moreover, the threats posed by sexual objectification victimization may be different for men and women (see Fischer et al., 2011 for a review) and, therefore, may not be equally addressed through sexual objectification perpetration. For women, sexual objectification may be associated with threats to physical safety and risks for sexual violence (Calogero et al., 2021; Gervais & Eagan, 2017). In contrast, objectification victimization among men may primarily threaten masculine norms of domination, power, competence, and sexual prowess (Pecini et al., 2023). Therefore, in response to objectifying experiences, men may especially seek to enact these masculine norms (Fagen & Anderson, 2012; Hlavka, 2017). Perpetrating sexual objectification may be seen as a more productive and effective way to address the threats faced by objectified men compared to those faced by objectified women because of its capacity to establish dominance (Bareket & Shnabel, 2020). Thus, we predicted that the relationship between sexual objectification victimization and perpetration would be stronger among men than women due to gender socialization and threats to masculinity.

As mentioned above, research thus far is somewhat limited to women as victims and men as perpetrators; however, some indirect evidence supports our prediction. Among women, sexual objectification victimization has been shown to predict antisocial behavior, such as retaliatory aggression (Poon & Jiang, 2020) and dishonesty (Poon et al., 2023). Because sexual objectification perpetration is another type of antisocial behavior and can be characterized as one form of aggression (Loughnan & Pacilli, 2014), this research indirectly supports our prediction that sexual objectification victimization may predict its perpetration.

Supporting our gender moderation prediction, men’s experiences of sexual victimization and objectifying experiences by women have been associated with feelings of emasculation (Fagen & Anderson, 2012; Hlavka, 2017). These feelings of emasculation have been shown to predict sexual objectification of women (Mikorski & Szymanski, 2017) and behaviors to regain control (Fagen & Anderson, 2012). One meta-analysis highlighted that dominance behaviors performed by women may incur social penalties compared to those enacted by men, given their divergence from feminine gender norms (Williams & Tiedens, 2016). While objectified women may be motivated to perpetrate sexual objectification to obtain benefits denied by their victimization, such as a sense of control (Dvir et al., 2021; Williams et al., 2017), the threat of social penalties may attenuate this response compared to among objectified men. Likewise, men’s greater social power may grant them the freedom to objectify others because high-power individuals have been shown to perpetrate more sexual objectification (Civile & Obhi, 2016). Altogether, we predicted that sexual objectification victimization would predict its perpetration and that this relationship would be moderated by gender, being stronger among men.

### Mediated Moderation Through Social Dominance Orientation

While moderation answers the question of when or for whom certain relationships exist or are stronger or weaker, mediation examines how or why a relationship occurs (Hayes, 2013). We proposed that SDO may be one mediator underlying our predicted gender moderation of the relationship between sexual objectification victimization and perpetration. Although researchers originally conceptualized SDO as a personality trait, studies have shown that it can be influenced by life experiences, such as social threat (Duckitt & Fisher, 2003) and childhood maltreatment (Teisl et al., 2012), and is better characterized as an attitude (Pratto et al., 2006). Despite this, relatively limited research has investigated how interpersonal situations may predict SDO. Given that SDO is associated with many negative outcomes, such as prejudice (Duckitt & Sibley, 2007; Stathi et al., 2021), reduced environmentalism (Stanley et al., 2019), and heightened conspiracy beliefs (Dyrendal et al., 2021), examining factors that predict SDO may offer insights for preventing these harmful outcomes.

According to social dominance theory, the preference for social hierarchy stems from the view that the world is competitive and that people utilize “ruthless and amoral” means to fulfill their needs and obtain advantages (Duckitt, 2001, p. 51). Sexual objectification emphasizes the instrumental use of the target as an object for the perpetrator’s sexual pleasure, disregarding the targets’ humanness and needs (Fredrickson & Roberts, 1997). Past research has highlighted that sexual objectification increases moral outrage and anger (Fagen & Anderson, 2012; Monachesi et al., 2023; Shepherd et al., 2023), hostile intent attributions (Poon & Jiang, 2020), and relative deprivation (Poon et al., 2023). These findings indirectly suggest that targets of sexual objectification may feel that they were subjected to ruthless, amoral acts for the perpetrator’s satisfaction. Meanwhile, other findings that sexually objectified targets utilize antisocial means, such as aggression and dishonesty (Poon & Jiang, 2020; Poon et al., 2023), may reflect their adoption of a competitive worldview whereby these antisocial behaviors are justified to make gains over others. Thus, sexual objectification victimization may predict SDO.

Because SDO is a preference for social hierarchies, it has been theorized to cause behaviors which maintain or reinforce these hierarchies (Pratto et al., 2006), such as prejudice (e.g., Duckitt & Sibley, 2007; Stathi et al., 2021) and intergroup violence (e.g., Rollero et al., 2021). Sexual objectification perpetration may be one means by which individuals assert their dominance (Bareket & Shnabel, 2020; Walton & Pedersen, 2022). Thus, individuals experiencing heightened SDO associated with objectification victimization may objectify others as a demonstration of power and a clear indicator that there are others below them within the social hierarchy (i.e., those who are only victimized) in a form of “last-place aversion” (Kuziemko et al., 2014).

However, research has found that SDO varies based on salient hierarchal contexts and group belonging (see Üzümçeker, 2023 for a review). After experiencing sexual objectification victimization, individuals may be primed to focus more on their gender and related gender hierarchies (Hundhammer & Mussweiler, 2012). Thus, sexual objectification victimization may relate to SDO differently among men and women. In particular, focusing on gender hierarchies following objectification victimization may predict greater SDO among men but not women because men benefit from the existing gender hierarchy whereas women do not (Rudman & Glick, 2021). In turn, SDO should significantly mediate the relationship between sexual objectification victimization and perpetration among men, not women, suggesting one path which may account for the proposed stronger relationship between victimization and perpetration among men in a mediated moderation (Muller et al., 2005).

This prediction has received empirical evidence from past research. One study found that, following a gender prime, men had significantly higher SDO than women, while nonprimed men and women did not have significantly different SDO levels (Walls, 2005). Gender also moderated the effect of a status manipulation on SDO, such that, when status was threatened, men showed higher SDO than women (Batalha et al., 2011). Given that sexual objectification may be one form of threat to one’s status, particularly men’s (Gervais et al., 2018), this empirical evidence lends indirect support to our prediction. Additionally, the link between SDO and sexual objectification perpetration has been robustly demonstrated. For example, men’s SDO was found to correlate with their objectification of women (Bareket & Shnabel, 2020); moreover, men with high SDO were more likely to objectify women when imagining being under their supervision (Bareket & Shnabel, 2020). Indirectly, SDO has been related to hostile sexism (Ruthig et al., 2021), as well as the tendency to aggress and use sex for self-advancement (Sinn & Hayes, 2018). Altogether, these findings support our prediction that SDO mediates the relationship between sexual objectification victimization and perpetration among men, but not women.

### Sexual Objectification Victimization, Gender, and Perceived Social Mobility

Research has highlighted the importance of individual factors in predicting SDO (Sibley & Duckitt, 2008) and sexual objectification perpetration (see Loughnan & Pacilli, 2014 for a review); therefore, the investigation of moderating factors allows for greater understanding of these complex phenomena. As mentioned above, perceived social mobility refers to individuals’ beliefs about their potential to change their social status within their society (Day & Fiske, 2019). Given that sexual objectification is closely tied to and often motivated by power and status (Bareket & Shnabel, 2020; Walton & Pedersen, 2022), one’s beliefs about the malleability of their social status may influence the relationship between sexual objectification victimization, gender, SDO, and sexual objectification perpetration.

According to the prospect of upward mobility hypothesis (Benabou & Ok, 2001), social mobility beliefs often relate to upward social mobility*—*the attainment of a higher social status. Because social status relies on the existence of hierarchies, individuals with higher perceived social mobility may prefer a society with clear social hierarchies to measure their status attainment and reap greater benefits when achieving this. Existing research indirectly supports this argument by demonstrating that perceived social mobility increases system defense and tolerance for inequality (Day & Fiske, 2017; Shariff et al., 2016), and decreases support for government spending on education (Wen & Witteveen, 2021). In the context of sexual objectification, benefits of social advancement may include avoiding future victimization.

As mentioned previously, objectification victimization may make the gender hierarchy salient (Hundhammer & Mussweiler, 2012). When gender is salient to men in relation to their objectification victimization, their status as the dominant group limits their potential for upward social mobility; however, they may seek to reaffirm this dominant status. Therefore, regardless of their perceived social mobility, objectification victimization may positively predict SDO among men. In contrast, when gender is made salient among women, perceived social mobility offers a chance for advancement and a means to reduce victimization experiences. For example, advancement in social class may represent opportunities to avoid sexually objectifying workplaces for women (Moffitt & Szymanski, 2011), but not for men. Similarly, physical attractiveness may represent opportunities to attain greater social power for women but not men (Fredrickson & Roberts, 1997), allowing women who feel they can control their attractiveness to perceive themselves as having social mobility (Wang et al., 2020). Thus, perceived social mobility should moderate the relationship between sexual objectification victimization and SDO and the indirect relationship between sexual objectification victimization and perpetration through SDO among women but not men. Specifically, women with greater perceived social mobility may show more support for social hierarchies, resembling the relationships among men, because they feel they are able to move upwards through them to avoid future objectification victimization. In contrast, women with lower perceived social mobility may show less support for such hierarchies because they feel restricted to inferior groups, instead favoring egalitarian ideals as a means to avoid future victimization.

These predictions have received empirical evidence from past research. One study found that men’s SDO was consistent, regardless of social status, after gender was made salient (Huang & Liu, 2005). In contrast, the same study found that women in higher status demographic groups showed greater SDO after gender priming compared to women lower in social status (Huang & Liu, 2005). This suggests that, when gender is salient, women may rely on other factors that grant them social power in determining their SDO. Another study found that increases in perceived social mobility, indicated by a traditionally subordinate political group winning a presidential election, led to increased SDO among that political group, while SDO among the traditionally dominant group was unchanged (Liu et al., 2008). This implicates the role of perceived potential for upward social mobility in determining SDO among those in a traditionally subordinate group, supporting our prediction.

### Current Study

In this research, we examined the relationship between sexual objectification victimization and perpetration, with a focus on how it differs among men and women. We predicted that these gender differences were due to differences in socialization related to social power within a patriarchal society (Rudman & Glick, 2021); therefore, we considered the roles of power-related variables to understand further nuance in this relationship. Specifically, we investigated the role of SDO as a mediator and perceived social mobility as a moderator. We proposed five hypotheses:

#### *H*1

Sexual objectification victimization positively predicts sexual objectification perpetration.

#### *H*2

Gender moderates the relationship between sexual objectification victimization and perpetration, such that it is stronger among men.

#### *H*3

SDO mediates the moderation by gender in *H*2 (see Fig. 1a), such that the mediation is only significant among men, but not women.

#### *H*4

A three-way interaction between sexual objectification victimization, gender, and perceived social mobility significantly predicts SDO. Specifically, perceived social mobility does not moderate the relationship between objectification victimization and SDO among men. Among women, victimization positively predicts SDO among those with high perceived social mobility and negatively predicts SDO among those with low perceived social mobility.

#### *H*5

The three-way interaction on SDO carries further implications for sexual objectification perpetration in a moderated mediated moderation (see Fig. 1b). Specifically, perceived social mobility does not influence the indirect relationship between sexual objectification victimization and perpetration through SDO among men. Women high in perceived social mobility show an indirect relationship between objectification victimization and perpetration through SDO, similar to men. The indirect relationship through SDO is not significant for women low in perceived social mobility.

We conducted a priori power analysis using G*Power 3.1.9.7 to calculate the required sample size (Faul et al., 2009). Based on research on sexual objectification (Holland et al., 2017; Poon & Jiang, 2020) and perceived social mobility (Bettencourt et al., 2001), we anticipated small-to-medium effect sizes (assuming *f* = 0.18). The results showed that a sample of 451 participants was required to detect the three-way interaction between objectification victimization, social mobility, and gender with power of 0.80 and alpha of 0.05. Thus, we aimed to recruit at least 451 participants.

## Method

### Participants

We recruited 533 participants from CloudResearch, a platform used to collect reliable data (Hauser & Schwartz, 2016). Three participants failed an attention check and, therefore, their responses were excluded from data analyses. Another four participants’ responses were excluded for missing data. Including the excluded responses would not influence the results. The final sample consisted of 526 participants (218 women, 308 men; *M*_age_ = 41.8 years, *SD* = 12.8). The final sample was 77.57% White, 9.70% Black, 6.27% Asian, 2.85% multiracial, 1.14% Native American (including South American), 0.38% Pacific Islander and Native Hawaiian, and 2.09% a race not listed. The present research received ethical approval from the institutional review board of the authors’ university, and we obtained informed consent from all participants prior to the study. Participants completed the measures below for a small monetary reward. The instructions assured participants that all responses were confidential, would not be used for any form of evaluation, and were only identifiable through anonymous codes to mitigate potential social desirability bias given the sensitive nature of the topics measured.

### Measures

#### Perceived Social Mobility

Participants completed the 6-item Subjective Social Mobility scale (Huang et al., 2017) to assess their perceived social mobility. Sample items include “According to the present society, I am capable of improving my social status” and “I could improve my social situation provided I strived constantly” (1 = *strongly disagree*, 7 = *strongly agree*). Responses were averaged with higher scores indicating greater perceived social mobility (α = 0.90).

#### Sexual Objectification Victimization

To assess participants’ sexual objectification victimization, they completed the 15-item Interpersonal Sexual Objectification scale (Kozee et al., 2007). Sample items include “How often have you heard a rude, sexual remark made about your body?” and “How often have you been touched or fondled against your will?” (1 = *never*, 5 = *almost always*). Participants responses were averaged with higher scores indicating more sexual objectification victimization (α = 0.95).

#### Social Dominance Orientation

Participants then completed the 4-item Short SDO scale (Pratto et al., 2013). Sample items include “In setting priorities, we must consider all groups” (R) and “Superior groups should dominate inferior groups” (1 = *extremely oppose*, 10 = *extremely favor*). Responses were reverse coded when needed and averaged with higher scores indicating greater SDO (α = 0.82).

#### Sexual Objectification Perpetration

We used the 15-item Interpersonal Sexual Objectification scale—Perpetration Version (Gervais et al., 2018) to assess participants’ frequency of perpetrating sexual objectification. Sample items include “How often have you made a rude, sexual remark about someone’s body?” and “How often have you touched or fondled someone against her/his will?” (1 = *never*, 5 = *almost always*). Responses were averaged with higher scores indicating greater sexual objectification perpetration (α = 0.94).

### Data Analysis Strategy

Data analysis was carried out using SPSS 28.0 with PROCESS, a regression path analysis modeling tool which offers templates for testing various mediation and moderation models (Hayes, 2013). Specifically, we used PROCESS models 1, 8, and 11 to test our predictions (Hayes, 2013). We adopted bootstrapping analyses with 5000 samples and used the 95% bias-corrected bootstrap confidence intervals (95% BCBCIs) to test for the existence of indirect relationships. The indirect relationship was considered to stably exist if the confidence interval did not include zero. Given that age and race may influence SDO and perceived social mobility (Assari, 2018; Zubielevitch et al., 2023), we controlled for these factors in our analyses.

## Results

We first calculated descriptive statistics and Pearson correlations for all variables of interest (see Table 1). All variables were also checked for normality and multicollinearity using their skewness, kurtosis, and variance inflation factor. The skewness was no greater than |1.80|, kurtosis no greater than |3.30|, and variance inflation factor no greater than 1.01. Therefore, there were no issues related to normality nor multicollinearity in the data and we could proceed with regression analyses (Thompson et al., 2017; Weston & Gore, 2006). Continuous predictor variables (i.e., sexual objectification victimization, perceived social mobility, and SDO) were mean centered for data analysis. Gender was coded as “0” for women and “1” for men.

**Table 1:** Descriptive statistics and Pearson correlations for all variables

	Mean	*SD*	*α*	1	2	3	4
*Whole Sample*							
1. Sexual objectification victimization	1.95	0.76	.95	1.00			
2. Perceived social mobility	4.11	1.41	.90	.00	1.00		
3. Social dominance orientation	3.53	2.14	.82	.03	.04	1.00	
4. Sexual objectification perpetration	1.72	0.72	.94	.42^***^	.20^***^	.29^***^	1.00
*Female*							
1. Sexual objectification victimization	2.28	0.67	.93	1.00			
2. Perceived social mobility	3.78	1.49	.92	.10	1.00		
3. Social dominance orientation	3.12	2.17	.85	− .06	0.03	1.00	
4. Sexual objectification perpetration	1.50	0.58	.93	.43^***^	.22^**^	.20^**^	1.00
*Male*							
1. Sexual objectification victimization	1.71	0.74	.96	1.00			
2. Perceived social mobility	4.35	1.31	.88	.07	1.00		
3. Social dominance orientation	3.82	2.08	.79	.20^***^	− .01	1.00	
4. Sexual objectification perpetration	1.88	0.77	.94	.65^***^	.12^*^	.29^***^	1.00

Means for all variables were significantly different between men and women

For variables 1 and 4, absolute range, 1-5; for variable 2, absolute range, 1-7; for variable 3, absolute range, 1-10

^*^p < .05, ^**^ *p* < .01, ^***^ *p* < .001, two-tailed

### H1

Sexual Objectification Victimization Predicts Sexual Objectification Perpetration

We predicted that sexual objectification victimization would positively predict sexual objectification perpetration. Simple regression analysis indicated that sexual objectification victimization significantly predicted perpetration in the full sample (*b* = 0.39, *SE* = 0.04, *p* < 0.001), supporting our hypothesis.

### H2

Gender Moderates the Relationship Between Victimization and Perpetration

We then tested whether gender would moderate the relationship between sexual objectification victimization and perpetration using PROCESS model 1 (Hayes, 2013). Sexual objectification victimization alone still significantly and positively predicted sexual objectification perpetration (*b* = 0.39, *SE* = 0.06, *p* < 0.001). Likewise, gender also significantly predicted sexual objectification perpetration (*b* = 0.68, *SE* = 0.05, *p* < 0.001), indicating that being male predicted greater perpetration than being female regardless of objectification victimization experiences. Importantly, the interaction between sexual objectification victimization and gender on sexual objectification perpetration (*b* = 0.29, *SE* = 0.07) was significant, *F*(1, 520) = 15.87, *p* < 0.001. Simple effect analysis revealed that the relationship between sexual objectification victimization and perpetration was significant among men and women, but was stronger among men (*b* = 0.68, *SE* = 0.04, *p* < 0.001) than women (*b* = 0.39, *SE* = 0.06, *p* < 0.001). Thus, our results supported *H*2.

### H3

Social Dominance Orientation Mediates the Objectification Victimization x Gender Interaction on Objectification Perpetration

To test whether SDO mediated the moderation effect of gender in the relationship between sexual objectification victimization and perpetration, we used PROCESS model 8 (Hayes, 2013). In the full sample, sexual objectification victimization alone did not significantly predict SDO (*b* = − 0.12, *SE* = 0.22, *p* = 0.579). However, gender significantly predicted SDO (*b* = 0.84, *SE* = 0.20, *p* < 0.001), indicating that being male predicted greater SDO than being female, regardless of objectification victimization experiences. Importantly, the interaction between objectification victimization and gender on SDO was also significant (*b* = 0.72, *SE* = 0.27, *p* = 0.007). Specifically, sexual objectification victimization significantly predicted SDO among men (*b* = 0.60, *SE* = 0.16, *p* < 0.001), but not among women (*b* = − 0.12, *SE* = 0.22, *p* = 0.579).

Sexual objectification perpetration was also significantly predicted by sexual objectification victimization (*b* = 0.40, *SE* = 0.06, *p* < 0.001), gender (*b* = 0.62, *SE* = 0.05, *p* < 0.001), and the interaction between sexual objectification victimization and gender (*b* = 0.24, *SE* = 0.07, *p* < 0.001). SDO also significantly and positively predicted sexual objectification perpetration (*b* = 0.06, *SE* = 0.01, *p* < 0.001).

Finally, the model supported our mediated moderation prediction (index of mediated moderation = 0.05, *SE* = 0.02) because the 95% BCBCI excluded 0 (0.012 to 0.085). Among men, SDO significantly mediated the relationship between sexual objectification victimization and perpetration (point estimate = 0.04, *SE* = 0.01) because the 95% BCBCI excluded 0 (0.019 to 0.059). In contrast, among women, SDO did not significantly mediate the relationship between sexual objectification victimization and perpetration (point estimate = − 0.01, *SE* = 0.02) because the 95% BCBCI included 0 (− 0.040 to 0.022).

### H4

Three-Way Interaction Predicts Social Dominance Orientation

To test for the three-way interaction between sexual objectification victimization, gender, and perceived social mobility in predicting SDO, we used PROCESS model 11 (Hayes, 2013) to test the model in Fig. 1b. The three-way interaction (*b* = − 0.39, *SE* = 0.18) was significant in predicting SDO (see Table 2), *F*(1, 516) = 4.63, *p* = 0.032. Among men, there was no interaction between sexual objectification victimization and perceived social mobility in predicting SDO (*b* = − 0.03, *p* = 0.816). Sexual objectification victimization consistently predicted SDO among men with high (1 *SD* above the mean) perceived social mobility (*b* = 0.57, *SE* = 0.21, *p* = 0.006) and low (1 *SD* below the mean) perceived social mobility (*b* = 0.65, *SE* = 0.27, *p* = 0.015).

**Table 2:** Relationship between sexual objectification victimization and social dominance orientation by perceived social mobility and gender

	Perceived Social Mobility	Effect	*SE*	*p*
Female	Low	− 0.536	0.266	.045
	High	0.494	0.322	.126
Male	Low	0.654	0.267	.015
	High	0.575	0.206	.006

Low and high perceived social mobility represent 1 *SD* below and above the mean, respectively, after mean centering

Among women, there was a significant interaction between sexual objectification victimization and perceived social mobility in predicting SDO (*b* = 0.36, *p* = 0.008). Specifically, sexual objectification victimization did not predict SDO among women with high (1 *SD* above the mean) perceived social mobility (*b* = 0.49, *SE* = 0.32, *p* = 0.126); however, for women with low (1 *SD* below the mean) perceived social mobility, sexual objectification victimization predicted less SDO (*b* = − 0.54, *SE* = 0.27, *p* = 0.044).

### H5

Implications of the Three-Way Interaction on Sexual Objectification Perpetration through SDO

For the overall mediation, the results of the same PROCESS model 11 (Hayes, 2013) that tested *H*4 indicated that the three-way interaction and SDO carried further implications for sexual objectification perpetration (index of moderated mediated moderation = − 0.04, *SE* = 0.02; see Table 3) because the 95% BCBCI did not include 0 (− 0.076 to − 0.002). As predicted, perceived social mobility did not moderate the mediation among men (index of conditional moderated mediation = 0.00, *SE* = 0.01) because the 95% BCBCI included 0 (− 0.025 to 0.015). The indirect relationship between objectification victimization and perpetration through SDO was significant among men high (1 *SD* above the mean) in perceived social mobility (point estimate = 0.05, *SE* = 0.02, 95% BCBCI [0.019 to 0.096]) and low (1 *SD* below the mean) in perceived social mobility (point estimate = 0.06, *SE* = 0.02, 95% BCBCI [0.024 to 0.114]).

**Table 3:** Indirect relationships between sexual objectification victimization and sexual objectification perpetration through social dominance orientation by perceived social mobility and gender

	Perceived Social Mobility	Point Estimate	Bootstrap* SE*	95% BCBCI
Female	Low	− 0.051	0.030	− 0.111, 0.005
	High	0.047	0.034	− 0.022, 0.110
Male	Low	0.062	0.022	0.024, 0.114
	High	0.055	0.019	0.019, 0.096

Low and high perceived social mobility represent 1 *SD* below and above the mean, respectively, after mean centering

Perceived social mobility significantly moderated the mediation among women (index of conditional moderated mediation = 0.03, *SE* = 0.02) with the 95% BCBCI excluding 0 (0.004 to 0.067). The mediation effects were not significant for women low in perceived social mobility (point estimate = − 0.05, *SE* = 0.03, 95% BCBCI [− 0.111 to 0.005]) nor for women high in perceived social mobility (point estimate = 0.05, *SE* = 0.03, 95% BCBCI [− 0.022 to 0.110]). However, pairwise contrasts showed that the mediation among women high in perceived social mobility did not differ significantly from the mediation among men in either high (95% BCBCI [− 0.058 to 0.085]) or low (95% BCBCI [− 0.096 to 0.054]) perceived social mobility group. Meanwhile, the mediation among women low in perceived social mobility was significantly different from that among women high in perceived social mobility (95% BCBCI [0.013 to 0.184]), as well as men high (95% BCBCI [0.037 to 0.184]) and low (95% BCBCI [0.043 to 0.196]) in perceived social mobility (i.e., all other groups; see Table 4 for details on pairwise contrasts). Thus, although the mediations among women did not reach significance, our predictions were still partially supported. When simultaneously considering the gender moderation of the direct path, this pattern of results remained (see Supplementary File for details).

**Table 4:** Pairwise contrasts of indirect relationships by perceived social mobility and gender

		Female		Male			
		High perceived social mobility		Low perceived social mobility		High perceived social mobility	
		Contrast	95% BCBCI	Contrast	95% BCBCI	Contrast	95% BCBCI
Female	Low perceived social mobility	0.098	0.013, 0.184	0.113	0.043, 0.196	0.105	0.037 to 0.184
	High perceived social mobility			− 0.015	− 0.096, 0.054	0.008	− 0.058, 0.085

Low and high perceived social mobility represent 1 *SD* below and above the mean, respectively, after mean centering; contrasts were determined to be significant if the 95% BCBCI excluded 0

## Discussion

Objectification theory proposed that women’s frequent experiences of sexual objectification may lead to the adverse effect of self-objectification, whereby targets of objectification internalize this experience and objectify themselves (Fredrickson & Roberts, 1997). However, the relationship between sexual objectification victimization and the perpetration of sexual objectification against others, to our knowledge, had not been explored. Moreover, gender differences in the consequences of victimization and antecedents of perpetration and their corresponding psychological mechanisms had not been investigated. We addressed these knowledge gaps by revealing that sexual objectification victimization predicted the perpetration of sexual objectification, that this relationship was stronger among men than women, and that it was mediated by SDO among men, but not among women.

Although the mediation was not significant among women, we demonstrated a significant three-way interaction between sexual objectification victimization, gender, and perceived social mobility such that perceived social mobility moderated the relationship between victimization and SDO among women, but not men. Among women with low perceived social mobility, the link between objectification victimization and SDO was significant and negative, suggesting that victimization may be associated with a preference for equality among this group. Among women with high perceived social mobility, the relationship had a substantial positive effect size, despite not being significant. This three-way interaction on SDO also carried implications for sexual objectification perpetration. For women with low perceived social mobility, the indirect relationship was negative and approaching statistical significance, indicating low SDO as a potential buffer against objectification perpetration. Like the direct relationship between victimization and SDO, the indirect relationship of victimization on perpetration through SDO among women with high perceived social mobility had a substantive positive effect size, despite not reaching significance. The failure to reach significance in this group may be due to the small number of women who self-perceived high social mobility (*n* = 27). Altogether, these findings highlight the crucial role of social power and socialization processes in determining gender differences in sexual objectification outcomes.

### Advancement of Existing Knowledge

The demonstration of the relationship between sexual objectification victimization and perpetration elaborates on previously identified consequences of victimization and antecedents of perpetration (see Loughnan & Pacilli, 2014 for a review). This finding was consistent with past research showing that interpersonal victimization may increase perpetration of the same form of maltreatment (e.g., bullying, workplace undermining, and sexual coercion; Falla et al., 2022; Lee et al., 2016; Russell & Oswald, 2001). The current study adds to this body of literature by identifying sexual objectification as another form of maltreatment where victimization may cyclically predict perpetration. Given the prevalence and dire outcomes of this phenomenon (Holland et al., 2017), identifying this relationship may present an opportunity to intervene and reduce the experience of sexual objectification.

While research on sexual objectification has often focused on gendered roles, primarily women as victims and men as perpetrators (e.g., Sáez et al., 2019), the present research also advances current knowledge by acknowledging and demonstrating greater complexity in these roles as both victimization and perpetration were investigated among men and women. We identified notable gender differences; specifically, that victimization among men (vs. women) more strongly predicted objectification perpetration, significantly predicted SDO, and significantly predicted perpetration indirectly through heightened SDO, and that these relationships were not influenced by men’s perceived social mobility.

Similarly, the present findings add to research on behaviors associated with SDO which, based on past research, include prejudice (Stathi et al., 2021) and intergroup violence (Rollero et al., 2021). The current findings examine sexual objectification perpetration, which may be considered one form of prejudice and intergroup violence most often perpetrated against women (Brinkman & Rickard, 2009), as another antisocial behavior associated with SDO. Moreover, we present SDO as one potential explanation for the gender differences in the relationship between sexual objectification victimization and perpetration being that it may exacerbate the relationship among men, but not women. This finding is consistent with past research which found that SDO predicts sexual objectification perpetration among men (Bareket & Shnabel, 2020). However, Bareket and Shnabel (2020) found that SDO did not predict women’s objectification of men. In contrast, we found that SDO predicted objectification perpetration among men and women. This difference may suggest that women’s SDO leads them to objectify other women, a phenomenon demonstrated in past research (Strelan & Hargreaves, 2005). This may present a novel way in which objectification victimization harms women as this experience may relate to increased objectification perpetration against women from men and women alike.

The present results also identify interpersonal factors that may predict SDO which had previously received less research attention. Past research had shown that childhood maltreatment (Teisl et al., 2012) and social threat (Duckitt & Fisher, 2003) may predict SDO; however, the current study highlights an everyday interpersonal experience as potentially important in determining SDO. In particular, we demonstrated that being targeted by sexual objectification may be related to SDO and highlighted how individual-level factors related to gender and perceived social mobility may influence this relationship. Establishing that SDO may be predicted by sexual objectification victimization and also predict objectification perpetration lends some preliminary support to the conceptualization of SDO as an attitude that is both a cause and a result of hierarchical relationships within a society (Pratto et al., 2006). Given that sexual objectification is related to power and dominance (Civile & Obhi, 2016; Walton & Pedersen, 2022), it may be one way that these hierarchical relationships are reflected and perpetuated in real life.

Moreover, the demonstrated three-way interaction advances existing knowledge of sexual objectification and how it is experienced by men and women. This interaction showed that perceived social mobility differentially influenced the relationship between sexual objectification victimization and SDO, as well as the indirect relationship between victimization and perpetration through SDO, among men and women. This was consistent with past research on social mobility and SDO whereby social mobility influenced the SDO of a traditionally subordinated group, but not a dominant group (Liu et al., 2008). Interestingly, perceived social mobility moderated the relationship between objectification victimization and SDO among women such that those with low and high perceived social mobility showed comparable effect sizes in opposite directions, with a negative relationship among those low in perceived social mobility, and a positive—though not significant—one among women high in perceived social mobility. Thus, women’s perceived social mobility showed qualitative differences in the relationship between sexual objectification victimization and support for group equality versus social hierarchy, offering different implications for sexual objectification perpetration.

Finally, the finding that the indirect relationship between sexual objectification victimization and perpetration through SDO among women with high perceived social mobility, despite not reaching statistical significance, did not significantly differ than that among men demonstrates the importance of social status and power in relation to sexual objectification. When women, a traditionally subordinated group, perceived themselves as possessing the ability to gain social power, gender differences in the indirect effect disappeared and they did not report behaving significantly differently than men, a traditionally dominant group. Thus, the findings offer novel empirical evidence of the centrality of socialization processes related to power and gender norms underlying gender differences in sexual objectification.

### Theoretical and Practical Implications

The present findings also offer important theoretical and practical implications for future consideration and application. The significance of the indirect relationship among men, but not women, suggests a need to investigate gender differences in the consequences of sexual objectification victimization, as well as the need to examine beyond gendered victim and perpetrator roles. Moreover, while demonstrated consequences may exist among both objectified men and women, their underlying motivations may vary, offering different implications for coping with such experiences. SDO among men may also underlie relationships between sexual objectification victimization and its outcomes previously demonstrated among women, such as predicting dishonesty (Poon et al., 2023) and increasing aggression (Poon & Jiang, 2020). These associations and consequences, to our knowledge, have yet to be studied in objectified men, and may be related through SDO.

Additionally, our results highlight the significance of perceived social mobility in predicting outcomes of sexual objectification victimization. Given that perceived social mobility may reflect perceptions of ability to reduce future victimization, it may be relevant in determining responses to other forms of interpersonal maltreatment. For example, the previously mentioned cycles of victimization related to bullying, undermining at work, and sexual coercion (Falla et al., 2022; Lee et al., 2016; Russell & Oswald, 2001) may be moderated by social mobility perceptions in a way that is theoretically similar to that proposed in the present research.

Practically, identifying factors which influence objectification perpetration may help to reduce this phenomenon and, in turn, lower the prevalence of victimization. SDO, which mediated the relationship between victimization and perpetration among men, is negatively predicted by empathy (Stathi et al., 2021). Therefore, interventions for increasing empathic concern may reduce the perpetration of sexual objectification by objectified men (see Trivedi-Bateman & Crook, 2022 for a review). For both men and women with high perceived social mobility, objectification victimization predicted greater objectification perpetration in the present findings. Past research has suggested that social status could be improved by offering value to a group rather than through intimidation, and that a preference for the former strategy could be primed (Case et al., 2018) or increased by life history strategies (Maner & Hasty, 2023). Thus, clinicians may seek to encourage this prosocial strategy of status improvement among individuals with higher perceived social mobility coping with sexual objectification victimization to reduce potential perpetration of sexual objectification.

On a larger scale, the identification of variables related to social power (i.e., SDO and perceived social mobility) in the relationship between sexual objectification victimization and perpetration shine a light on the need to address socialization processes which promote this. As mentioned in the introduction, gendered power differentials (Rudman & Glick, 2021) and differences in gender norms (Pecini et al., 2023) may account for the differences seen in the present research between men and women. Thus, changes in cultural messaging which reinforce these may help to mitigate the relationship between objectification victimization and perpetration. For example, cultural messaging through media may influence sexual and gender socialization (Dill & Thill, 2007; Endendijk et al., 2022); thus, the present findings may motivate policymakers, activists, and content creators to implement changes to promote more positive socialization and reduce harmful cultural messaging.

### Limitations and Future Research Directions

Although our predictions were supported, there remain limitations which offer routes for future research. Firstly, some of the measures used may lack the specificity needed to capture further nuance in the demonstrated relationship. For example, we sought to understand sexual objectification beyond gendered victim and perpetrator roles, leading to a lack of gender specificity in the measures. While research has highlighted female objectification by men (Sáez et al., 2019), it remains unclear whether men’s victimization was by women, gay men, or another group entirely. Masculine ideals often center around sexual prowess with women and a disdain for homosexuality (Iacoviello et al., 2022), thus the perpetrator of sexual objectification against men may influence the proposed model. Likewise, it is unclear if women’s perpetration was against men, other women, or another group. Past research suggested that women objectify men and other women (Gervais et al., 2018; Strelan & Hargreaves, 2005), and that women’s SDO is associated with hostile sexism towards women (Ruthig et al., 2021) but not the objectification of men (Bareket & Shnabel, 2020). Therefore, future research may use more specific measures and alternative methods to develop understanding of the relationships between sexual objectification victimization, SDO, and sexual objectification perpetration among men and women.

Similarly, the SDO measure did not mention specific groups. Research has demonstrated that people consider real-world social inequalities when responding to SDO items (Schmitt et al., 2003), suggesting that women’s responses reflect a preference for men’s dominance. However, the moderation effect of perceived social mobility in the link between victimization and SDO among women may be due to a preference for other social hierarchies. For example, women from privileged racial or socioeconomic groups may perceive greater social mobility and endorse hierarchies related to these factors, rather than gender, to attain upward social mobility and avoid future victimization. Alternatively, women with high perceived social mobility may show a preference for gender inequalities favoring women (Schmitt et al., 2003). Future research may investigate the specific hierarchies that are supported following objectification victimization.

Additionally, the use of cross-sectional methodology limits our capacity to draw causal conclusions or determine the temporal ordering of the tested relationships. This is particularly relevant given that the proposed moderated mediated moderation model is somewhat complex. The present findings are only preliminary; however, they do highlight important relationships between sexual objectification victimization, perceived social mobility, SDO, and sexual objectification perpetration among men and women. Future research using more rigorous research methods, such as longitudinal or experimental research, is essential to examine the temporal ordering of the variables tested, determine the causal nature of the proposed relationships, and to offer greater reliability to the present findings through conceptual replication. The use of experimental designs may also allow perceived social mobility to be manipulated to create more equal sample size distributions given that the small sample of women who perceived high social mobility may explain why some relationships failed to achieve statistical significance in the present study. Additionally, the present results may have been influenced by social desirability bias in reporting SDO and sexual objectification perpetration given their antisocial nature. Future research may better account for this and adopt measures to control for social desirability biases.

Finally, the present research investigated one mechanism that may account for the gender differences in the relationship between sexual objectification victimization and perpetration, specifically the stronger relationship among men compared to women. The significance of SDO in mediating this relationship among men but not women may emphasize the central role of gender socialization and patriarchy in determining objectification victimization outcomes and perpetration predictors. However, we did not examine other potential mechanisms that may explain this relationship uniquely among women or universally regardless of gender. While SDO stems from competitive worldviews, the highly related ideology of right-wing authoritarianism stems from dangerous world beliefs (Duckitt & Fisher, 2003). Given that sexual objectification may be viewed by women as particularly dangerous (Calogero et al., 2021; Fischer et al., 2011; Gervais & Eagan, 2017), victimization may predict perpetration through authoritarianism rather than SDO among women. Likewise, among men and women, self-objectification has been robustly found to be predicted by objectification victimization (e.g., Davids et al., 2019; Holland et al., 2017) and demonstrated to predict the objectification of others (Strelan & Hargreaves, 2005). Thus, future research may investigate self-objectification as a mechanism underlying the relationship between sexual objectification victimization and perpetration among men and women, which may be insightful for further development of objectification theory (Fredrickson & Roberts, 1997).

### Conclusion

Sexual objectification is a harmful experience frequently targeting women. Thus far, research has been relatively restricted to focusing on women’s role as its victims and men’s role as its perpetrators, with these perspectives often considered in isolation. The present research addressed this by demonstrating that sexual objectification victimization predicts sexual objectification perpetration and that this relationship was stronger among men than women. We also revealed SDO as a mediator and perceived social mobility as a moderator. Our moderated mediated moderation revealed that the direct and indirect relationships among women with high perceived social mobility resembled those among men, such that perceived social mobility reduced gender differences. In contrast, gender differences persisted among women with low perceived social mobility compared to men. Altogether, the present model offers a nuanced understanding of sexual objectification, acknowledging complex victim—perpetrator roles and individual differences, which may aid in the development of programs for coping with and preventing such experiences.

## Supplementary Information

Below is the link to the electronic supplementary material.

Supplementary file1 (DOCX 29 KB)

## Data Availability

All data and materials are available upon reasonable request.
