# Supplementary material for: Gendered Cycles of Sexual Objectification: The Roles of Social Dominance Orientation and Perceived Social Mobility
Source: Arch Sex Behav. 2024 Dec 19;54(2):657–71. doi: 10.1007/s10508-024-03065-3 (PMC11836084; doi:10.1007/s10508-024-03065-3)
Supplement: Supplementary file 1 — Supplementary file1 (DOCX 29 KB) [file 10508_2024_3065_MOESM1_ESM.docx]

**Overall Model**

To simultaneously test for the three-way interaction between sexual objectification victimization, gender, and perceived social mobility and the gender moderation of the direct relationship between sexual objectification victimization and perpetration (see Figure S1), we used PROCESS model 13 (Hayes, 2013). Gender significantly moderated the direct relationship between sexual objectification victimization and perpetration (*b* = 0.24, *SE* = 0.07), *F*(1, 519) = 11.71, *p* < .001, *ΔR^2^* = .013. When accounting for this, the results indicated that the three-way interaction on SDO still carried significant implications for sexual objectification perpetration (index of moderated mediated moderation = −0.02, *SE* = 0.01; see Table S1) because the 95% BCBCI did not include 0 (−0.052 to −0.001). The indirect relationship between sexual objectification victimization and perpetration through SDO was significant for men, regardless of their perceived social mobility. In contrast, the indirect relationship between sexual objectification victimization and perpetration through SDO was not significant for women, regardless of their perceived social mobility (see Table S1). According to the pairwise contrasts, the mediation among women with high perceived social mobility did not differ significantly from either group of men; whereas, the mediation among women with low perceived social mobility was significantly different from all other groups (see Table S2). This was consistent with the results reported in the main results.

**Table S1**

*Indirect* *Relationships Between Sexual Objectification Victimization and Perpetration Through Social Dominance Orientation by Perceived Social Mobility and Gender After Accounting for Gender Moderation of Direct Relationship*

|  | Perceived Social Mobility | Point Estimate | Bootstrap SE | 95% BCBCI |
| --- | --- | --- | --- | --- |
| Female | Low | −0.034 | 0.021 | −0.079, 0.004 |
|  | High | 0.031 | 0.023 | −0.016, 0.075 |
| Male | Low | 0.041 | 0.015 | 0.015, 0.075 |
|  | High | 0.036 | 0.013 | 0.011, 0.062 |

*Note.* Low and high perceived social mobility represent 1 *SD* below and above the mean, respectively, after mean centering.

**Table S2**

*Pairwise Contrasts of Indirect Relationships* *by Perceived Social Mobility and Gender After* *Accounting for Gender Moderation of Direct Relationship*

|  |  | Female | | Male | | | |
| --- | --- | --- | --- | --- | --- | --- | --- |
|  |  | High perceived social mobility | | Low perceived social mobility | | High perceived social mobility | |
|  |  | Contrast | 95% BCBCI | Contrast | 95% BCBCI | Contrast | 95% BCBCI |
| Female | Low perceived social mobility | 0.065 | 0.006, 0.134 | 0.075 | 0.028, 0.135 | 0.070 | 0.023, 0.128 |
|  | High perceived social mobility |  |  | −0.010 | −0.036, 0.063 | 0.005 | −0.040, 0.053 |

*Note*. Low and high perceived social mobility represent 1 *SD* below and above the mean, respectively, after mean centering; contrasts were determined to be significant if the 95% BCBCI excluded 0.

**Figure S1**

*Conceptual Model*

Sexual objectification perpetration

SDO

Social mobility

Sexual objectification victimization

Gender
